# Stomathognatic system function in indigenous people from Brazilian Xingu villages: An electromyographic analysis

**DOI:** 10.1371/journal.pone.0243495

**Published:** 2020-12-15

**Authors:** Carla Moreto Santos, Marcelo Palinkas, Wilson Mestriner-Júnior, Isabela Hallak Regalo, Paulo Batista de Vasconcelos, Fernando José Dias, Jaime Eduardo Cecilio Hallak, Selma Siéssere, Simone Cecilio Hallak Regalo

**Affiliations:** 1 Department of Basic and Oral Biology, Ribeirão Preto School of Dentistry, University of São Paulo, São Paulo, Brazil; 2 Department of Nursing, Faculty Anhanguera, Ribeirão Preto, Brazil; 3 Department of Neuroscience and Behavioral Sciences, Faculty of Medicine of Ribeirão Preto, University of São Paulo and National Institute and Technology - Translational Medicine (INCT.TM), Ribeirão Preto - São Paulo, Brazil; 4 Department of Integral Adults Dentistry, Research Centre in Dental Sciences (CICO), Dental School, Universidad de La Frontera, Temuco, Chile; UCSI University, MALAYSIA

## Abstract

The maintenance of postural balance can be influenced by the lifestyle of a population. This study aimed to determine the electromyographic activity of the masseter and temporalis muscles during mandibular tasks and habitual and non-habitual chewing in indigenous individuals to reveal the differences among white Brazilian individuals. Sixty Brazilians (18 and 28 years) were divided into two groups: 30 Xingu indigenous individuals and 30 white Brazilian individuals, with 20 men and 10 women in each group. The individuals were assessed using the normalized electromyographic activity of mandibular tasks (rest, protrusion, right and left laterality) and electromyographic activity of masticatory cycles in habitual (peanuts and raisins) and non-habitual (Parafilm M) chewing. Data were analyzed using Student’s t-test (p < .05). Comparisons between the groups demonstrated significant differences. Indigenous individuals group presented a decrease in the normalized electromyographic activity of the masticatory muscles during mandibular rest [right masseter (p = .002) and left masseter (p = .004) muscles]. There was increase in the normalized electromyographic activity during protrusion [left temporal (p = .03) muscle]. There was increase in the electromyographic activity during chewing: peanuts [right masseter (p = .001), left masseter (p = .001) and right temporal (p = .01) muscles], raisins [right masseter (p = .001), left masseter (p = .002), right temporal (p = .008), left temporal (p = .01) muscles] and Parafilm M [left masseter muscle (p = .05)]. From the findings of this study, we concluded that in the comparison between indigenous and white individuals, positive changes were observed in the electromyographic pattern of the masticatory muscles in the mandibular postural conditions, with greater masticatory efficiency in the indigenous group.

## Introduction

The modern-day diet is based on the consumption of soft, industrialized foods, which contain an immense amount of sugar, high sodium concentration, and very few nutrients [1, 2]. These factors can affect the stomatognathic system, mainly due to the ingestion of soft foods, which provides less vigorous chewing, causing possible functional changes in the biomechanics of the masticatory muscles [3].

Factors such as eating habits, dentition status, malocclusion, teeth morphology, and symptoms of temporomandibular disorders play a role in the masticatory system that can lead to functional changes [4]. Indigenous people have general habits appropriate to human health and ideal for the proper functioning of the masticatory muscular system [5]. The satisfactory nutritional status and daily routines of these Natives are part of their cultural identity and allow the preservation of healthy lifestyle habits [6].

The food of the Brazilian indigenous population living in the Xingu National Park is poorly processed, without spices, basically composed of roots and dried fruits, red meat from wild animals, and fish [7]. However, the Brazilian white population has a diet based mainly on soft and processed foods that can lead to hypofunction of the complex interrelationship between the morphology and biomechanics of facial and masseter and temporalis muscles [8, 9].

Thus, the study of indigenous populations that still preserve part of their hunter-gatherer and natural lifestyle is a useful comparison when analyzing the effects of lifestyles adopted by the white urban society on the masticatory performance. This understanding can help identify if the lifestyle of a population has made a real contribution to dysfunctions of the stomatognathic system observed in white urban populations and can be helpful for the design of treatments for this problem.

Our null hypothesis was that differences exist between patterns of electromyographic activity of the masseter and temporal muscles at indigenous individuals from Brazilian Xingu villages with Brazilian urban white people.

## Material and methods

### Ethical approval

This study was approved by the Research Ethics Committee at the Ribeirão Preto School of Dentistry, University of Sao Paulo (# 2004.1.747.58.6), and by the Xingu Indigenous Council.

### Study population

A post hoc analysis was conducted assuming a significance level of 0.05 and a power of 96%, with an effect size f of 0.90 for comparison of right masseter muscle for chewing with peanuts, and the sample size calculation indicated that the required number of individuals is 30 for each group. The sample size calculation was performed with G*Power software v 3.0.10 (Franz Faul, Kiel University, Kiel, Germany).

Following the t-test inclusion criteria, 60 individuals, normal occlusion (Angle Class I) were selected to participate in this study and were divided into two groups: Xingu indigenous Brazilians, age between 15 and 45 years (mean 23.6 years) and white urban Brazilians, age between 17 and 46 years (mean 22.8 years). There were 10 females and 20 males in each group. Individuals were paired one-to-one (gender and age). The Brazilian federal government protects this indigenous population. Therefore, they are confined to an area of the Xingu National Park and have no contact with unauthorized white individuals. A translator was responsible for the communication between the researchers in this study and the indigenous people.

The diet of the Xingu indigenous Brazilians is based on low-processed foods, such as roots and dried fruits; red meat from wild animals, like monkeys, sloths, wild pigs; reptile meat, like alligators and lizards; and fish. The white population has a diet considered soft and processed based on rice or processed pasta, cooked beans, vegetables (mostly cooked).

The inclusion criteria for the study are as follows: complete dentition; normal occlusion; no neurological, psychiatric, or movement disorders; no reports of toothaches; having satisfactory periodontal health; absence of extensive facial skeletal alterations; and no previous treatments using occlusal splints. The clinical signs and symptoms of temporomandibular disorders were evaluated using clinical examinations and questionnaires, based on the Research Diagnostic Criteria for TMD Axis I [10, 11].

Participants were excluded if prosthetic devices or orthodontic braces were used; had neurological, psychiatric or movement disorders; mixed dentition; or a history of oral/maxillofacial surgery or treatment for temporomandibular disorders.

### Electromyographic analysis

The evaluation of the electromyographic activity of the masseter and temporalis muscles was performed on all individuals using a MyosystemBr1 (DataHominis Ltd., Uberlândia, Minas Gerais, Brazil) apparatus. Indigenous individuals were examined in their tribes in the Xingu National Park, whereas white participants were tested at the Laboratory of Electromyography of the Ribeirão Preto Dentistry School at the University of São Paulo, Brazil.

Differential active sensors were placed on the center of the masseter and temporalis muscles belly, between the innervation zone and the distal tendon, on the motor point [12]. The position of the sensors was determined by palpation, and they were fixed with adhesive tape, with the length of the bars perpendicular to the muscle fibers, according to the recommendations of Surface EMG for Non-Invasive Assessment of Muscles (SENIAM) [12]. The skin was cleaned with alcohol a few minutes before the surface electrodes were positioned to reduce impedance [13]. A circular stainless steel electrode (three centimeters in diameter) was used as a reference sensor (ground electrode) fixed on the skin of the frontal bone region.

Electromyographic activity was evaluated during mandibular rest (5s), protrusion, dental clenching in maximum voluntary contraction with (4s) and without (4s) inert material, right (5s) and left (5s) laterality. Each mandibular task was performed three times and the average of these values was used for the study.

The records of the electromyographic signals of the masticatory cycles were measured by the mathematical calculation of the ensemble averaged linear envelope during the habitual chewing with peanuts (consistency hard) (10s) and raisins (consistency soft) (10s), and non-habitual chewing of inert material (10s). The inert material consisted of a folded paraffin sheet (Parafilm M^®^, Pechinery Plastic Packaging, Batavia, IL, USA; 18×17×4 mm; weight 245 mg) inserted between the occlusal surfaces of the first and second molars on the right and left side [14].

In non-habitual chewing, the hinge-type short excursion movement occurred. This type of chewing standardizes the movement and reduces the effects of the change in length x muscle tension, typical occurrences for dynamic records. Each chewing process was performed only once.

To calculate the results obtained from the ensemble averaged linear envelope of the chewing cycles of the electromyographic signal, the first three initial chewing cycles were eliminated, maintaining the central cycles, because in the initial phase of the chewing process, the initial cycles show a variation in the movement pattern of the jaw.

The individual’s postural position was respected during the collection of electromyographic data, where everyone remained seated, with their feet resting on the ground, palms resting on their thighs and their necks erect, determining Frankfurt’s horizontal plane parallel to the floor.

The values of RMS and ensemble averaged linear envelope of the electromyographic signals were normalized by the amplitude values obtained with the average of three maximum voluntary contractions of the masticatory muscles, obtained during dental clenching.

### Statistical analysis

The electromyographic data were normalized and statistically analyzed (Statistical Package for the Social Sciences Version 22.0 for Windows, IBM Inc.; Chicago, IL, USA). The statistical analyses were performed using one-way analysis of variance. Confidence level set at 5% (p < .05) to test for significance (means, lower, upper and ± SE values).

## Results

Normalized electromyographic values for each muscle studied at each clinical condition for the two groups are shown in Table 1. There was significant difference between groups with a decrease in the normalized electromyographic data during mandibular rest [right masseter (p = .002) and left masseter (p = .004) muscles] for the indigenous individuals group. There was increase in the normalized electromyographic activity during protrusion [left temporal (p = .03) muscle] for the indigenous individuals group.

**Table 1:** Mean, standard error (SE), lower (L), upper (U), and statistical significance of normalized electromyographic data during the mandibular taks in the groups: Xingu indigenous Brazilians group (G1), and white urban Brazilian group.

	Groups						p value
	GI			GII			
Mandibular Task	mean (SE)			mean (SE)			
Muscles		L	U		L	U	
*Rest*							
Right masseter	0.03(0.004)	0.01	0.09	0.07(0.01)	0.02	0.25	.002*
Left masseter	0.03(0.005)	0.01	0.14	0.05(0.004)	0.01	0.13	.004*
Right temporal	0.08(0.01)	0.02	0.49	0.08(0.01)	0.02	0.24	NS
Left temporal	0.09(0.01)	0.02	0.40	0.09(0.01)	0.02	0.32	NS
*Protrusion*							
Right masseter	0.16(0.02)	0.02	0.47	0.21(0.03)	0.02	0.91	NS
Left masseter	0.15(0.02)	0.02	0.51	0.19(0.02)	0.05	0.64	NS
Right temporal	0.13(0.02)	0.03	0.44	0.09(0.01)	0.02	0.39	NS
Left temporal	0.15(0.02)	0.03	0.71	0.08(0.01)	0.02	0.40	.03*
*Right Laterality*							
Right masseter	0.12(0.02)	0.01	0.78	0.10(0.02)	0.01	0.68	NS
Left masseter	0.10(0.01)	0.01	0.37	0.14(0.01)	0.03	0.37	NS
Right temporal	0.13(0.02)	0.02	0.81	0.12(0.01)	0.04	0.35	NS
Left temporal	0.12(0.02)	0.02	0.52	0.08(0.01)	0.02	0.43	NS
*Left Laterality*							
Right masseter	0.14(0.02)	0.01	0.51	0.13(0.01)	0.02	0.59	NS
Left masseter	0.08(0.01)	0.01	0.41	0.07(0.007)	0.01	0.17	NS
Right temporal	0.11(0.02)	0.01	0.81	0.09(0.01)	0.02	0.28	NS
Left temporal	0.15(0.02)	0.03	0.61	0.11(0.01)	0.03	0.53	NS

*significant difference, NS: not significant, one-way analysis (i.e., p < .05)

The normalized electromyographic data for habitual (peanuts and raisins) and non-habitual (Parafilm M) chewing for the groups are shown in Table 2. Differences were found between groups in the habitual chewing (peanuts) in the right (p = .001) and left (p = .001) masseter muscles and right temporal muscle (p = .01); habitual chewing (raisins) in the right (p = .001) and left (p = .002) masseter muscles and right (p = .008) and left (p = .01) temporalis muscles; and non-habitual chewing (Parafilm M) in the left masseter muscle (p = .05) with increase in the electromyographic activity at indigenous individuals as compared with white Brazilian individuals.

**Table 2:** Mean, standard error (SE), lower (L), upper (U), and statistical significance of normalized electromyographic data during the habitual and non-habitual chewing in the groups: Xingu indigenous Brazilians group (G1), and white urban Brazilian group.

	Groups						p value
	GI			GII			
Chewing	mean (SE)			mean (SE)			
Muscles		L	U		L	U	
*Peanuts*							
Right masseter	0.47(0.06)	0.16	1.76	0.76(0.05)	0.19	1.47	.001*
Left masseter	0.43(0.03)	0.14	0.78	0.70(0.05)	0.23	1.47	.001*
Right temporal	0.48(0.05)	0.17	1.53	0.64(0.03)	0.38	1.34	.01*
Left temporal	0.57(0.05)	0.22	1.33	0.69(0.05)	0.37	1.74	NS
*Raisins*							
Right masseter	0.33(0.03)	0.06	0.81	0.31(0.04)	0.11	1.15	.001*
Left masseter	0.31(0.04)	0.05	1.43	0.51(0.03)	0.14	1.17	.002*
Right temporal	0.36(0.04)	0.06	1.51	0.51(0.02)	0.13	0.99	008*
Left temporal	0.41(0.03)	0.19	1.06	0.55(0.03)	0.14	1.10	01*
*Parafilm M*							
Right masseter	0.53(0.06)	0.13	1.97	0.70(0.05)	0.33	1.64	NS
Left masseter	0.46(0.04)	0.10	1.03	0.58(0.03)	0.21	1.13	.05*
Right temporal	0.55(0.07)	0.12	1.84	0.63(0.05)	0.15	1.95	NS
Left temporal	0.59(0.07)	0.13	1.69	0.63(0.04)	0.16	1.47	NS

*significant difference, NS: not significant, one-way analysis (i.e., p < .05)

## Discussion

The present study investigated the presence of differences in the electromyographic patterns of two distinct populations. The differences found in this study were attributable probably due to different lifestyles between the groups, that is, Xingu indigenous group maintained a traditional lifestyle connected with its culture, whereas the world modernization process influenced the white Brazilian group.

Several studies have analyzed the electromyographic activity of masticatory muscles in different situations of urban populations such as age, sex, temporomandibular dysfunction, chronic degenerative diseases, dentition status, and oral parafunctional habits [15–18], but there is a gap in the literature that does not show the masticatory muscle behavior of specific populations with eating habits of native villagers, such as indigenous populations.

In the present investigation, electromyographic activity was recorded during mandibular rest conditions in the masseter and temporalis muscles in two groups, indicating that lower activity of the masseter muscles in the Xingu indigenous.

The indigenous population in Xingu has carefully preserved life habits transmitted from one generation to the next that might somehow contribute to the development of healthy individuals [19, 20].

During the assessment of the maintenance of rest, it can be argued that Brazilian whites have interferences in the functioning of the stomatognathic system that promote greater electromyographic activity when compared to the indigenous individuals. Studies state that the increased electrical activity of the muscle during rest is considered a sign of disharmony in the stomatognathic system [21, 22].

The change in the electromyographic activity of the masticatory muscles may be associated with occlusal interferences such as premature contacts that cause imbalances in the stomatognathic system [23], justified by the biofeedback of the information captured by the fibers of the periodontal ligament and the nervous system [24]. In this study, it was not assessed whether the white Brazilian group had premature dental contacts.

Our results showed that during the protrusion condition, both groups exhibited a contraction pattern to maintain postural position, with greater activation of the masseter muscles when compared to the temporalis muscles. Higher electromyographic recordings in the temporalis muscles were found in the Xingu indigenous group, with a significant difference for the left temporal muscle.

In the right and left laterality conditions, greater electromyographic activity is expected to be recorded in the temporalis muscles on the same side. The jaw extends to (work side), whereas for the masseter muscle greater contralateral activity is to be expected [25].

It was evident that our result did not follow the normality pattern of neuroanatomical muscle activation in the right laterality condition in which larger electromyographic records were expected in the masseter muscle on the contralateral side of the mandible for the Xingu indigenous group.

The factor that may have influenced the incorrect neuroanatomical movement in this clinical condition would be the difficulty of communication with the indigenous village, sometimes making it impossible to understand the mandibular movement [26, 27]. In the clinical condition of the left laterality, the neuroanatomic pattern was correct in both groups.

Fibrous and rigid feeding throughout life promotes important changes in the masticatory system, which is a characteristic of indigenous tribes, providing occlusal wear, which is a mechanism of physiological adjustment in the masticatory system, which influences the neuroanatomical pattern [28–30]. Several authors defend the opinion that this type of wear is useful for strengthening the periodontium of the teeth with the increased electromyographic activity of the masticatory muscles in mandibular postural conditions [31]. In this study, we observed greater electromyographic activity of the masseter and temporalis muscles in almost all clinical mandibular conditions.

The function of the lateral pterygoid muscles that present activation of their fibers arranged horizontally during protrusion and mandibular excursion movements [32], can be compromised by occlusal factors, such as interferences and dental wear, progressively overloading the other masticatory muscles, modifying the neuroanatomical pattern. This factor can also modify the neuroanatomical pattern in the clinical conditions of protrusion and laterality of the mandible.

The analysis of the electromyographic data recorded during habitual and non-habitual chewing provided evidence of muscle performance. The comparison of the data obtained from the two groups during masticatory activities revealed lower electromyographic activity for all muscles in the group of indigenous individuals.

The diet of the indigenous population of the Xingu has a diet rich in proteins with essential amino acids for the formation of nervous and muscular structures, and the abundance of fiber and hard food consumption that favors efficient training for the stomatognathic system [33, 34]. In this way, synchrony, and power of all the components that make up this system are achieved, which allows the best performance during its function.

The absence of daily stress may also have provided some training throughout the life of indigenous individuals, which led the fibers of the masseter and temporalis muscles of these individuals to have the best possible performance. This optimal function enables them to grind hard foods all day long without wearing out their muscles, at the same time that they recruit a minimum of motor units and muscle fibers to perform this task [14, 35].

The habitual (peanuts and raisins) and non-habitual (Parafilm M) chewing revealed the capacity of the indigenous participants’ stomatognathic system to contract the muscles involved as little as possible. In the chewing process of the Xingu indigenous group, more motor units were spared the effort to grind the food, thus favoring muscle health with greater masticatory efficiency concerning white Brazilian individuals. However, the stomatognathic system of white Brazilian individuals may have stopped receiving natural stimuli to develop, not adapting to dietary changes, due to the global industrialization that produced soft and super caloric foods [36, 37].

Considering that electromyographic activity of the masseter and temporalis muscles on the mandibular tasks and chewing (habitual and non-habitual) was significantly different in the comparison between groups, our null hypothesis was not rejected.

This study was carried out in a totally isolated environment and with a population of principles and customs different from the white population. These facts made that with each contact with the indigenous population, there was an awareness process, being necessary to wait for the moment that the indigenous population accepted the data collection procedure.

There are limitations to the electromyographic analysis of the masseter and temporalis muscles. The difficulty of understanding the Portuguese language, by the indigenous population, of what would be the movement of a mandibular clinical condition assessed in electromyographic activity may have interfered in the data collection, even having a local translator in the indigenous village. Among the thirty indigenous assessed in this study, one third did not speak Portuguese and did not even understand the language.

## Conclusions

The indigenous people from Brazilian Xingu villagers showed a reduction in electromyographic activity in mandibular tasks and an increase in masticatory efficiency. These results are strong indications that dietary and culture influences, structure and activity of masticatory muscles, oral habits, proprioceptive input from periodontium /muscles during chewing, wear of occlusal surfaces (due to diet) and lifestyle interfere with the functioning of the stomatognathic system and that the ancient wisdom and tradition of the indigenous population enable the maintenance of healthy masticatory system functioning.

## Supporting information

S1 File Data from the 30 individuals of the Xingu indigenous group. (PDF)

S2 File Data from the 30 individuals of the white Brazilian group. (PDF)
