# Supplementary material for: Stomathognatic system function in indigenous people from Brazilian Xingu villages: An electromyographic analysis
Source: PLoS One. 2020 Dec 15;15(12):e0243495. doi: 10.1371/journal.pone.0243495 (PMC7737974; doi:10.1371/journal.pone.0243495)
Supplement: S1 File — (PDF) [file pone.0243495.s001.pdf]

Xingu indigenous Brazilians	Rest				Protr	
	RM	LM	RT	LT	RM	LM
Aimare	2.78	2.42	3.43	2.87	7.98	4.76
Araci	4.65	3.55	6.91	5.25	35.41	35.08
Caindo	8.02	3.02	5.47	31.12	24.65	3.69
Katita	3.23	2.98	18.59	5.72	5.56	5.52
Natuiu	3.97	3.97	3.04	5.77	18.05	23.26
Porï	4.42	4.11	9.48	12.40	55.34	41.04
Reateayup	4.45	6.17	6.92	3.23	12.06	13.94
Tainan	4.03	4.65	10.98	7.50	21.74	34.83
Tamem	7.05	3.55	4.51	5.11	49.67	37.32
Yuwipo	6.39	6.28	4.61	16.68	23.12	9.45
Amtoti	5.02	4.48	11.16	22.49	6.99	4.90
Ariup	4.53	7.68	10.51	13.24	50.93	2.82
Arutá	3.41	6.75	2.85	7.36	22.68	48.46
Atai	4.31	3.77	7.38	5.74	7.28	5.09
Itacaré	4.70	2.50	6.21	6.97	46.79	44.36
Juat	3.89	11.49	8.92	8.20	12.16	9.32
Kleber	3.82	3.32	5.26	7.82	4.70	5.97
Mekïrïpo	3.06	3.25	4.89	4.19	13.59	17.26
Melobo	3.37	4.06	7.17	6.98	5.60	5.75
Moyup	3.11	4.19	10.07	3.25	36.46	12.85
Ngaty	3.26	4.07	3.44	4.25	24.52	24.41
Pablo	3.92	3.15	6.05	12.06	45.05	45.43
Piwara	2.70	3.61	18.92	5.41	52.84	133.10
Pnewo	3.66	3.88	4.00	3.99	13.30	10.40
Rogério	8.29	6.95	10.89	6.24	12.87	29.81
Tafút	3.77	4.03	3.77	5.40	14.56	5.53
Tapi	5.16	4.12	7.87	7.63	7.27	40.98
Upit	2.51	2.26	3.80	6.30	9.88	6.72
Yanin	3.37	3.31	4.90	3.74	17.46	13.18
Yukwarï	3.60	3.69	3.28	4.52	65.99	52.13

usion		Right Laterality				Left La	
**RT**	**LT**	**RM**	**LM**	**RT**	**LT**	**RM**	**LM**
4.20	4.47	3.66	3.37	3.98	4.16	3.43	3.49
8.87	14.16	29.21	33.59	24.52	14.27	35.21	8.15
6.42	16.27	54.32	3.79	6.01	12.07	85.08	3.92
8.41	13.17	37.53	7.55	8.27	19.61	3.58	3.90
4.82	9.22	4.13	5.11	3.39	5.80	7.15	5.99
31.89	21.64	29.08	40.36	7.48	13.14	7.20	26.65
10.03	3.95	3.28	5.25	6.24	3.59	6.08	6.28
18.38	20.24	5.36	10.82	16.32	15.53	5.70	21.34
30.97	20.18	36.76	15.08	9.04	6.00	10.41	6.52
23.32	43.98	10.67	8.36	15.21	32.05	16.81	7.91
13.47	18.66	5.16	7.67	13.09	20.52	6.41	4.23
10.26	13.63	30.36	2.55	10.76	13.58	35.02	2.61
3.88	6.45	23.37	15.80	10.60	8.02	35.03	39.54
9.76	27.60	6.15	3.71	5.93	30.14	5.49	3.77
4.67	3.48	12.40	28.89	11.78	3.54	11.32	12.56
8.56	11.57	14.24	6.94	6.86	11.14	15.17	7.08
4.37	6.03	4.13	11.49	6.40	5.50	5.15	3.92
6.08	8.29	3.38	6.41	8.54	12.71	10.65	10.42
5.49	5.95	9.02	7.83	4.60	5.18	8.70	6.35
9.57	5.41	3.74	7.22	11.23	4.76	9.45	4.14
7.67	8.59	9.70	28.22	18.47	7.40	20.09	10.68
5.50	10.69	15.45	34.81	45.76	10.97	39.31	26.78
18.52	6.39	5.56	14.16	12.60	4.76	49.59	67.90
4.35	5.11	6.59	10.17	4.20	5.35	7.66	5.01
24.23	14.78	5.70	22.73	8.34	6.48	42.02	10.54
4.96	12.00	5.51	8.48	7.08	13.70	13.30	3.94
6.60	8.01	7.33	9.30	13.13	6.58	7.28	4.76
12.81	5.17	27.01	9.05	11.06	6.68	37.43	19.77
12.40	8.69	7.46	9.62	7.95	6.50	12.45	9.09
9.74	11.84	49.91	61.00	21.55	4.83	58.67	20.36

terality		Chewing peanuts				Chewin	
RT	LT	RM	LM	RT	LT	RM	LM
4.50	4.46	88.34	114.49	38.67	37.08	66.05	28.48
12.00	36.59	39.27	79.82	31.18	120.45	49.49	18.89
7.29	21.18	60.38	36.26	23.78	30.04	23.60	17.82
7.14	7.23	22.73	67.93	42.97	82.35	39.24	31.51
3.49	6.05	76.77	70.01	67.59	35.27	50.78	40.13
4.96	9.71	58.74	51.41	40.76	67.92	41.49	27.34
7.28	3.53	43.76	83.88	48.24	64.44	32.18	71.56
12.58	10.45	31.62	30.56	39.32	37.38	32.75	33.18
5.35	5.56	46.88	19.48	17.43	17.46	84.88	32.08
10.64	37.72	24.36	39.01	31.34	37.90	18.89	17.19
11.99	17.77	80.44	61.41	33.24	55.95	27.26	129.67
12.08	15.73	40.94	34.54	17.08	26.89	20.07	27.60
5.02	11.14	36.17	66.72	51.11	26.33	50.39	137.27
10.40	18.36	68.14	29.92	45.77	111.12	53.12	16.54
10.13	9.39	328.42	52.96	74.21	64.95	75.80	45.75
7.33	9.08	75.86	41.18	64.65	77.28	64.94	36.23
4.28	6.55	53.65	92.80	61.57	70.74	51.44	26.41
5.27	38.30	57.58	117.08	42.83	59.66	85.68	72.27
3.89	5.70	64.27	21.29	29.70	23.10	27.99	14.66
8.90	8.21	41.98	93.23	50.86	81.87	16.45	19.50
6.39	13.38	117.69	70.42	38.01	37.31	99.48	60.39
8.97	19.13	57.68	192.25	59.69	98.85	46.40	92.24
10.58	7.50	44.00	72.57	32.37	38.02	22.57	26.56
4.00	4.41	67.96	70.10	53.27	44.38	39.25	41.52
15.64	10.14	73.46	113.01	60.72	50.71	31.62	46.57
8.15	12.18	45.38	98.30	32.68	38.22	22.90	97.06
3.96	8.03	48.33	45.72	89.64	35.11	22.75	39.85
11.91	4.96	64.16	41.56	63.55	90.03	44.56	30.28
6.08	4.83	69.95	99.13	63.33	43.11	45.48	57.30
21.35	24.90	64.65	39.21	26.07	32.39	36.82	31.84

g raisins		Chewing Parafilm M				ntal clenching in maxi	
RT	LT	RM	LM	RT	LT	RM	LM
33.08	20.40	146.10	118.75	68.93	45.97	323.18	239.49
24.28	87.02	63.86	55.95	36.77	71.68	247.03	241.45
10.77	17.35	72.96	73.00	27.47	33.82	180.41	151.64
51.14	67.67	35.03	57.27	61.52	57.73	48.19	92.09
48.90	17.00	53.51	54.06	47.40	27.23	72.28	98.39
20.62	29.15	21.87	15.74	15.21	27.29	174.22	164.61
44.89	57.56	14.74	33.64	13.19	18.11	101.10	146.23
32.94	36.51	22.30	25.05	36.17	32.70	46.46	89.01
31.96	25.61	116.95	68.16	41.20	45.18	199.31	74.47
17.27	32.54	28.80	46.14	11.97	29.87	80.85	91.09
24.69	39.98	100.95	92.39	46.74	47.44	269.68	258.84
9.88	28.36	99.50	66.33	55.55	66.24	109.08	154.57
55.55	35.57	118.83	69.27	86.33	70.92	140.94	95.66
38.50	25.71	54.64	28.07	54.16	41.40	306.87	179.34
23.79	22.45	70.68	34.59	32.83	25.97	275.39	130.17
46.34	63.86	88.89	53.71	70.63	88.25	197.91	107.54
52.18	37.60	58.16	83.48	42.07	51.32	75.28	119.22
24.66	50.94	143.84	120.42	60.66	73.94	251.98	164.08
22.14	18.52	72.92	30.53	41.54	28.69	36.52	29.78
8.36	117.32	69.78	51.55	43.77	112.31	264.92	250.26
31.81	28.33	147.68	136.65	71.35	65.56	193.77	150.77
39.88	66.01	50.78	64.55	65.80	60.09	195.44	295.52
25.12	23.27	48.13	113.06	44.69	48.76	210.19	502.91
43.35	30.60	72.17	73.17	72.67	62.21	114.84	125.38
30.15	35.09	44.96	70.27	43.14	40.67	215.08	309.77
21.08	34.42	94.00	124.76	57.11	63.45	149.60	255.32
81.84	28.46	54.94	80.22	98.21	43.30	149.74	194.53
31.58	48.56	85.43	24.57	63.54	100.02	73.85	77.04
49.75	37.85	50.54	85.47	50.36	46.31	133.66	210.42
12.38	23.47	116.37	124.12	60.88	61.79	183.30	166.59

mum voluntary contract	
**RT**	**LT**
134.56	88.17
111.35	171.38
79.08	77.59
91.73	84.61
161.30	85.61
115.60	101.47
112.49	135.91
57.07	53.61
69.98	62.63
79.43	62.04
151.18	134.95
98.73	114.96
122.24	121.34
155.28	85.68
94.65	87.11
99.18	178.75
84.33	90.16
113.76	177.04
14.64	17.40
137.12	206.93
63.69	38.78
165.65	200.32
126.93	120.74
147.13	119.68
139.53	161.36
39.36	41.42
194.10	124.13
164.91	175.16
133.04	80.25
26.49	52.47
