# Supplementary material for: Stomathognatic system function in indigenous people from Brazilian Xingu villages: An electromyographic analysis
Source: PLoS One. 2020 Dec 15;15(12):e0243495. doi: 10.1371/journal.pone.0243495 (PMC7737974; doi:10.1371/journal.pone.0243495)
Supplement: S2 File — (PDF) [file pone.0243495.s002.pdf]

White urban Brazilian	Rest				Protr	
	RM	LM	RT	LT	RM	LM
Adriana	3.87	4.54	3.58	7.35	3.90	4.54
Aline	4.38	4.23	19.84	10.47	20.81	8.39
Ana Paula	5.01	7.01	5.33	18.54	8.02	7.59
Bárbara	6.72	5.33	3.99	18.22	6.87	7.80
Daniela	3.88	5.71	11.04	5.81	18.87	58.73
Fernanda	6.06	5.77	6.89	22.39	13.83	47.05
Flaviane	23.08	5.50	4.36	6.04	19.88	39.38
Gisele	4.37	5.86	7.66	6.66	5.12	10.13
Karina	13.22	10.65	9.43	15.57	18.23	24.18
Maria	17.42	4.41	4.77	4.49	11.54	7.83
Alex	1.78	3.27	3.45	3.19	1.78	7.19
André	4.55	4.48	11.46	6.71	14.60	13.60
Breno	4.60	6.83	10.22	5.16	15.06	10.95
Cássio	13.48	5.00	5.77	11.16	33.76	14.83
Diogo	5.09	5.65	17.61	3.60	50.97	32.77
Eduardo	4.92	6.35	13.64	4.21	22.70	23.32
Felipe	5.17	5.44	4.17	15.62	9.63	14.30
Fernando	3.98	9.70	3.02	4.22	12.92	37.04
Fernando	3.75	2.70	5.08	3.92	13.12	13.84
Filipe	2.53	6.29	8.26	14.29	5.05	7.62
Flávio	3.94	4.34	20.16	3.99	33.46	45.17
João	7.94	9.70	7.29	5.72	7.00	9.07
Lucas	3.47	2.88	7.84	8.08	68.66	71.91
Marcos	2.85	2.88	3.25	3.39	8.93	30.14
Maurílio	4.71	5.05	6.11	10.55	61.87	38.91
Paulo	6.47	9.83	5.27	5.76	37.16	19.94
Petrus	5.03	4.82	6.61	11.71	38.55	7.01
Vinicius	6.38	10.80	9.13	6.15	7.70	27.65
Wilian	3.21	3.06	21.43	8.51	13.19	20.90
Wilson	3.52	4.23	4.58	6.69	34.41	25.77

usion		Right Laterality				Left La	
RT	LT	RM	LM	RT	LT	RM	LM
3.58	7.35	21.15	8.86	24.71	16.24	8.56	6.56
12.87	4.51	6.65	8.80	16.61	9.87	8.02	13.47
4.54	18.21	5.36	7.42	5.56	17.45	6.78	6.57
3.68	12.77	5.94	5.35	5.94	13.48	6.17	5.26
20.18	11.21	18.35	35.12	34.35	3.02	12.68	7.80
10.48	19.16	8.46	55.64	12.48	17.66	10.29	13.25
8.03	5.07	12.30	14.08	7.39	5.30	20.40	6.12
5.31	8.07	9.72	14.64	8.10	7.99	5.00	4.35
15.21	19.88	9.20	14.66	13.23	21.24	10.86	9.12
4.17	4.85	7.40	11.87	5.05	5.49	10.13	4.74
3.20	3.47	1.80	7.51	3.58	3.31	1.79	4.33
6.99	5.47	4.28	13.71	9.08	5.04	11.91	11.46
10.68	4.92	6.11	10.90	9.41	4.98	18.70	4.82
4.15	33.26	9.90	14.39	5.42	37.97	22.54	5.65
24.57	8.63	4.60	19.05	22.57	5.57	32.74	3.09
26.38	3.85	8.62	9.25	23.49	3.67	8.20	4.22
4.61	6.49	5.70	17.09	7.50	7.37	22.32	5.08
2.90	3.95	5.04	21.93	6.85	4.92	9.16	8.05
4.46	4.44	7.48	13.13	6.28	4.44	16.74	4.36
8.05	13.42	4.44	6.90	24.50	12.93	6.23	15.44
17.08	3.19	4.85	16.50	24.41	2.77	4.15	5.57
5.75	8.32	14.08	9.58	9.37	5.87	7.53	9.05
8.63	4.45	3.02	22.12	8.75	4.34	20.83	2.81
3.05	3.04	4.40	29.00	5.91	2.55	9.41	11.00
5.98	14.18	22.18	16.57	5.69	8.47	18.98	8.58
8.20	9.09	6.70	20.26	8.43	7.84	11.65	10.59
6.30	14.86	4.47	5.58	15.33	18.13	21.25	4.82
8.42	5.48	6.53	8.17	6.76	5.79	6.55	8.04
19.67	16.73	8.98	10.49	20.64	13.16	12.15	5.53
4.70	6.23	4.22	12.55	15.09	6.34	15.73	4.21

terality		Chewing peanuts				Chewin	
RT	LT	RM	LM	RT	LT	RM	LM
15.73	16.41	70.94	83.10	66.33	86.26	76.20	81.04
15.45	8.43	53.14	67.79	69.78	176.71	44.57	53.96
4.52	17.81	40.95	66.22	56.45	88.64	35.18	58.23
3.74	19.80	141.75	76.09	37.50	51.60	115.52	73.07
15.79	14.41	42.58	69.56	86.52	108.18	54.46	56.19
7.69	20.58	115.92	155.27	74.75	101.76	91.54	131.85
6.88	9.09	115.66	113.49	87.97	154.93	102.35	102.01
3.80	7.88	62.77	46.66	76.94	117.96	45.92	46.38
11.18	25.97	33.33	49.02	52.99	62.84	20.60	25.02
4.56	5.70	69.30	63.17	36.88	38.86	50.02	45.54
3.58	3.38	43.42	39.32	31.61	38.18	40.46	40.52
6.79	7.95	65.65	60.20	40.85	35.25	44.89	42.03
9.93	6.63	98.85	94.74	69.07	75.11	72.91	86.91
4.13	34.56	81.62	98.25	64.35	122.91	43.98	45.16
18.40	4.87	152.29	82.94	72.61	112.45	121.13	77.04
14.89	22.76	51.51	57.52	46.54	64.59	41.44	40.80
5.06	7.33	27.45	25.20	19.17	31.57	16.68	17.91
2.90	4.27	51.74	73.65	21.52	25.20	47.38	81.51
4.69	5.06	117.85	109.16	32.11	33.61	58.72	23.00
8.55	14.21	20.01	27.06	121.67	119.88	21.54	26.77
18.08	17.73	58.11	59.46	82.65	67.35	50.06	46.96
4.98	8.19	44.01	71.07	41.80	68.50	38.87	51.84
8.80	5.73	165.93	131.98	119.63	137.63	107.87	119.44
2.70	4.56	73.14	86.71	79.79	86.06	48.37	26.59
8.30	10.05	47.70	61.87	71.11	84.66	25.40	35.42
8.46	8.80	60.89	63.60	133.59	114.61	52.22	58.11
8.61	13.89	71.89	55.26	88.96	108.92	62.05	42.14
5.14	5.53	100.31	203.46	54.50	105.08	79.91	110.82
17.33	21.22	45.20	49.82	53.27	44.20	35.77	29.93
12.77	13.82	112.86	86.26	77.34	58.91	37.04	40.44

g raisins		Chewing Parafilm M				Dental clenching in ma[illegible]	
RT	LT	RM	LM	RT	LT	RM	LM
70.34	87.90	79.48	78.59	81.36	104.23	66.03	69.28
59.46	127.37	37.13	40.60	56.86	107.04	76.43	96.66
56.77	81.82	40.57	61.27	61.97	89.80	56.24	72.66
32.22	51.78	131.37	56.15	37.85	54.25	286.88	173.05
53.28	68.43	35.18	52.80	58.88	45.84	58.55	94.54
66.40	84.37	121.18	184.48	71.65	98.59	154.83	242.29
81.89	142.40	119.61	111.72	87.46	153.82	157.30	146.75
63.58	124.34	48.71	52.14	77.44	86.95	69.70	82.13
39.16	36.60	50.43	42.27	76.85	72.28	61.16	82.91
31.92	30.26	52.71	41.66	35.28	32.76	68.72	78.40
32.59	42.42	38.87	39.99	34.08	40.60	79.70	85.58
36.33	30.58	62.39	52.49	51.50	39.83	53.72	65.87
61.37	67.22	43.01	47.78	43.35	53.21	102.27	122.45
37.56	69.74	56.95	52.26	46.58	85.96	79.72	69.66
61.92	58.62	127.93	100.20	59.05	70.92	192.03	138.67
36.96	52.37	67.36	87.02	72.87	107.51	59.39	115.94
17.08	26.12	55.62	30.62	25.67	41.50	146.36	78.21
22.95	28.68	57.57	86.76	29.14	38.18	71.59	105.53
20.82	13.85	94.58	77.14	31.73	34.06	117.71	90.87
89.51	114.35	24.26	28.20	93.29	107.88	74.54	90.08
76.23	74.69	69.02	69.64	99.91	81.81	154.11	160.11
37.81	58.26	54.38	69.36	51.32	80.48	86.64	119.96
95.26	132.63	102.56	62.93	92.60	121.97	201.60	264.71
17.36	18.61	45.47	26.24	20.25	20.48	106.62	126.04
46.08	55.72	42.65	42.40	62.20	70.61	32.42	61.11
113.41	100.49	49.61	60.34	106.34	90.61	75.66	118.70
78.08	125.11	67.78	44.57	94.47	129.93	134.78	77.31
57.19	99.57	143.69	114.47	77.74	143.67	87.49	187.66
43.73	37.93	75.66	73.87	70.01	52.72	199.29	218.91
45.37	47.61	50.01	53.38	52.24	48.67	110.07	92.52

ximum voluntary contraction	
RT	LT
113.83	131.67
85.34	230.35
111.66	128.20
86.66	114.28
97.24	62.15
124.15	159.42
145.34	281.53
116.62	129.74
39.47	49.29
56.98	105.69
63.57	84.88
56.46	54.39
138.91	111.51
56.54	126.48
88.67	111.29
121.60	137.11
47.17	70.83
43.27	62.95
55.28	69.04
155.76	228.44
180.80	168.84
84.63	122.17
177.35	129.91
135.42	131.50
97.60	105.91
223.69	207.29
170.85	149.91
81.34	140.05
141.94	100.96
97.09	84.90
